# Time to Benefit of Sodium-Glucose Cotransporter-2 Inhibitors Among Patients With Heart Failure

**DOI:** 10.1001/jamanetworkopen.2023.30754

**Published:** 2023-08-24

**Authors:** KangYu Chen, Zhiqiang Nie, Rui Shi, Dahai Yu, Qi Wang, Fang Shao, Guohong Wu, Zhenqiang Wu, Tao Chen, Chao Li

**Affiliations:** 1 Department of Cardiology, The First Affiliated Hospital of USTC, Division of Life Sciences and Medicine, University of Science and Technology of China, Hefei, China; 2 Guangdong Cardiovascular Institute, Global Health Research Center, Guangdong Provincial People’s Hospital (Guangdong Academy of Medical Sciences), Southern Medical University, Guangzhou, China; 3 Heart Rhythm Centre, National Heart and Lung Institute, The Royal Brompton and Harefield National Health Service Foundation Trust, Imperial College London, London, United Kingdom; 4 Primary Care Centre Versus Arthritis, School of Medicine, Keele University, Keele, United Kingdom; 5 Department of Biostatistics, School of Public Health, Nanjing Medical University, Jiangsu, Nanjing, China; 6 Department of Geriatric Medicine, The University of Auckland, Auckland, New Zealand; 7 Center for Health Economics, University of York, York, United Kingdom; 8 Department of Epidemiology and Health Statistics, School of Public Health, Xi’an Jiaotong University Health Science Centre, Xi’an, China

## Abstract

**Importance:**

Emerging evidence has consistently demonstrated that sodium-glucose cotransporter 2 (SGLT2) inhibitors reduce the risk of heart failure (HF) hospitalization and cardiovascular (CV) death among patients with HF. However, it remains unclear how long a patient needs to live to potentially benefit from SGLT2 inhibitors in this population.

**Objectives:**

To estimate the time to benefit from SGLT2 inhibitors among patients with HF.

**Design, Setting, and Participants:**

This comparative effectiveness study systematically searched PubMed for completed randomized clinical trials about SGLT2 inhibitors and patients with HF published until September 5, 2022; 5 trials with the year of publication ranging from 2019 to 2022 were eventually included. Statistical analysis was performed from April to October 2022.

**Intervention:**

Addition of SGLT2 inhibitors or placebo to guideline-recommended therapy.

**Main Outcomes and Measures:**

The primary outcome was the time to first event of CV death or worsening HF, which was broadly comparable across the included trials.

**Results:**

Five trials consisting of 21 947 patients with HF (7837 [35.7%] were female; mean or median age older than 65 years within each trial) were included. SGLT2 inhibitors significantly reduced the risk of worsening HF or CV death (hazard ratio [HR], 0.77 [95% CI, 0.73-0.82]). Time to first nominal statistical significance (*P* < .05) was 26 days (0.86 months), and statistical significance was sustained from day 118 (3.93 months) onwards. A mean of 0.19 (95% CI, 0.12-0.35) months were needed to prevent 1 worsening HF or CV death per 500 patients with SGLT2 inhibitors (absolute risk reduction [ARR], 0.002). Likewise, 0.66 (95% CI, 0.43-1.13) months was estimated to avoid 1 event per 200 patients with SGLT2 inhibitors (ARR, 0.005), 1.74 (95% CI, 1.07-2.61) months to avoid 1 event per 100 patients (ARR, 0.010), and 4.96 (95% CI, 3.18-7.26) months to avoid 1 event per 50 patients (ARR, 0.020). Further analyses indicated a shorter time to benefit for HF hospitalization and among patients with diabetes or HF with reduced ejection fraction.

**Conclusions and Relevance:**

In this comparative effectiveness research study of estimating the time to benefit from SGLT2 inhibitors among patients with HF, a rapid clinical benefit in reducing CV death or worsening HF was found, suggesting that their use may be beneficial for most individuals with HF.

## Key Points

**Question:**

What is the time to benefit of sodium-glucose cotransporter 2 (SGLT2) inhibitors in individuals with heart failure?

**Findings:**

This comparative effectiveness research study consisting of 21 947 patients with heart failure found that the benefit of SGLT2 inhibitors first reached statistical significance at 26 days and sustained from 3.93 months onwards.

**Meaning:**

This study’s findings found a rapid clinical benefit of SGLT2 inhibitors, suggesting that their use may be beneficial for most individuals with heart failure.

## Teaser

This comparative effectiveness research study estimates the time to benefit from sodium-glucose cotransporter 2 (SGLT2) inhibitors among patients with heart failure.

## Introduction

Despite the significant advances in therapies, heart failure (HF) remains to be a global public health problem with a high risk for mortality, hospitalization, and poor quality of life. Emerging evidence has consistently demonstrated that sodium-glucose cotransporter 2 (SGLT2) inhibitors significantly reduce the risk of HF hospitalization and cardiovascular (CV) death among patients with HF and preserved ejection fraction (HFpEF) or reduced ejection fraction (HFrEF).^1,2,3,4,5^ These findings have been incorporated into the 2021 European Society of Cardiology guidelines for diagnosing and treating acute and chronic HF^6^ and then the 2022 American Heart Association/American College of Cardiology/Heart Failure Society of America guideline for the management of HF.^7^

Patients with chronic HF are at high risk for adverse prognosis with a 1-year postdischarge mortality rate ranging between 20% and 30% and the risk for readmission of approximately 20% to 25% at 1 month and approximately 50% at 6 months.^8^ Physicians are asked to focus on the sequencing of drug treatments and are advised to titrate each drug to the target dose for patients with HF.^9,10^ Additionally, patients with HF are often characterized by multiple chronic diseases or geriatric conditions, which tend to have polypharmacy. They may be exposed to immediate adverse events from drugs but experience delayed drug benefits. Therefore, to further support clinical treatment decisions, it is also crucial to acknowledge the time needed until drug treatments become substantially effective, apart from knowing the existence of treatment benefits.

Previously, the timing of the onset of treatment benefit was estimated by visually identifying the time point at which the curves separate but were subject to visual bias.^11,12^ In 2013, Lee et al^13^ proposed a framework for individualizing prevention decisions in older adults that incorporates the intervention’s lag time to benefit (TTB). For patients with a life expectancy shorter than the TTB, the use of medicines may pose the up-front harms associated with the interventions to older adults, with little chance that they survive long enough to receive the drug benefit.

Since then, few studies have estimated the TTB for statins therapy,^14^ blood pressure treatment,^15^ and bisphosphonate therapy.^16^ Unlike these treatments with a long delay between initiation of treatment and clinical benefit (eg, 1 year after antihypertensive treatment), SGLT2 inhibitors may implicate early immediate clinical outcomes due to their early physiologic changes among patients with HF. As such, in our current study, we conducted this analysis to estimate the TTB of SGLT2 inhibitors, as a whole drug class, based on individual participant data from completed randomized clinical trials (RCTs).

## Methods

### Institutional Review Board and Patient Consent

The Xi’an Jiaotong University Health Science Centre institutional review board (IRB) approved this study. The patient consent requirement was waived by the IRB because this was a secondary data analysis based on publications.

### Design

This comparative effectiveness research study used secondary data sets based on randomized clinical trials. To ensure the recent results could accurately reflect effectiveness of SGLT2 inhibitor therapies, we followed the International Society for Pharmacoeconomics and Outcomes Research (ISPOR) reporting guideline and addressed issues of framing the research question and reporting and interpreting findings.

### Data Source and Searches

This study was performed based on up-to-date published research. To ensure the completeness of including all SGLT2 inhibitors, we did a systematic review of the literature. Two independent reviewers (Q.W. and G.H.W.) searched relevant RCTs in PubMed that were published until September 5, 2022. Both reviewers screened titles and abstracts, followed by full texts, and a third reviewer (K.Y.C.) cross-checked the screening decision.

The search strategy is illustrated in the eAppendix in Supplement 1 following the previous systematic review and meta-analysis.^17,18^ In the present analysis, we only included RCTs comparing SGLT2 inhibitors vs placebo on CV events, death or HF hospitalization among patients with HF, regardless of the presence of type 2 diabetes. To serve the purpose of calculating TTB, we included studies having vector Kaplan-Meier (KM) curves, which enabled us to reconstruct individual time-to-event data from the number of patients at risk and the KM graph. Finally, we identified 636 articles from PubMed. Of them, 449 articles were excluded for the following reasons: nonhuman research (n = 32), meta-analysis or review (n = 181), not RCT study (n = 110), others (eg, letter, commentary) (n = 126). Among the 187 remaining studies, we identified 5 trials for the present analysis after excluding RCTs in patients without HF (n = 33), RCTs without CV outcome (n = 30), articles for the post hoc or secondary analysis (n = 90), studies for protocol or trial baseline articles (n = 27), and trials^19,20^ incapable of data reconstruction (n = 2) (eFigure 1 in Supplement 1).

### Outcome

The primary outcome in this analysis was the time to first event of CV death or worsening HF (HF hospitalization and urgent HF visit), which was broadly comparable across our included trials (eTable 1 in Supplement 1). Secondary outcomes included CV death, all-cause mortality, and hospitalization for HF were explored in our analysis.

### Data Reconstruction

We reconstructed individual time-to-event data in line with our previous publication through a 2-stage process.^15^ First, the quality data coordinates (survival probability and time) were extracted from KM curves by DigitizeIt software version 2.5 following the instructions from Liu and Lee.^21^ In stage 1, we also followed the recommendation when extracting data points. For example, extract as many points as possible and make sure the data points extracted are evenly distributed on the KM curves. Second, a Stata function (ipdfc command) developed by Wei and Royston^22^ was used to rebuild the individual data based on the aforementioned extracted raw data of time and survival probability. The algorithm underpinning the ipdfc command has been successfully used in our previous study,^15^ and basically aimed to estimate the number of censorings, the number of events, the censoring time, and the event time. We found that this algorithm recovered individual participant data from published trials with a high degree of accuracy (see eFigures 2, 3, 4, 5, and 6 in Supplement 1).

### Statistical Analysis

The characteristics of included studies were summarized from publications. The cumulative rates of primary outcome at each time point in the placebo and SGLT2 inhibitors group from the pooled trials were estimated using the KM curve. The hazard ratios (HRs) and their 95% CIs were calculated using the stratified Cox proportional hazards model to adjust for the clustering of patients from the same trial. We also calculated pooled HRs and 95% CIs using study-level meta-analysis to further estimate the efficacy of SGLT2 inhibitors. Meanwhile, heterogeneity between included studies was evaluated using the χ^2^ and *I*^2^ tests. The aforementioned analysis was repeated for secondary outcomes (ie, first hospitalization for HF, CV death, and all death)

To explore the timing for the first or sustained onset of clinical benefit of SGLT2 inhibitors (statistical significance at nominal *P* < .05), we calculated the HRs and 95% CIs for the treatment effect of SGLT2 inhibitors, with the data set truncated and iteratively reanalyzed in incremental cuts at each day. Furthermore, we fitted Weibull survival curves to estimate the time to specific absolute risk reduction (ARR) thresholds (ie, 0.002, 0.005, 0.010, and 0.020) using the conventional frequentist method to calculate the TTB and Monte Carlo simulations to derive its 95% CI. The detail of the calculation has been reported in our previous publication.^15^ We further presented TTB estimations by the following characteristics: individual trials; trials with different types of SGLT2 inhibitors (dapagliflozin, empagliflozin, or sotagliflozin); participants with or without diabetes; HF participants with a mild reduced/preserved ejection fraction or reduced ejection fraction. Statistical analysis was performed from April to October 2022. The TTB calculation was conducted in R version 3.4.0 (R Project for Statistical Computing), and other analyses in this study were performed in Stata version 15.0 (StataCorp).

## Results

The design and details of the 5 included RCTs have been reported previously and the study characteristics are summarized in Table 1. All trials were assessed as high quality with a low risk of bias across the 5 trials (eTable 2 in Supplement 1). Among the 21 947 participants in the 5 trials, 7837 (35.7%) were female, and the mean or median age within each trial was older than 65 years. The SGLT2 inhibitors were significantly better than the placebo in all 5 trials with a higher ARR for sotagliflozin (eFigure 2 in Supplement 1).

**Table 1.** Characteristics of Included Studies

Characteristic	DAPA-HF,^3^ 2019		EMPEROR-Reduced,^4^ 2020		EMPEROR-Preserved,^1^ 2021			SOLOIST-WHF,^2^ 2021		DELIVER,^5^ 2022	
	Dapagliflozin	Placebo	Empagliflozin	Placebo	Empagliflozin	Placebo		Sotagliflozin	Placebo	Dapagliflozin	Placebo
No. of countries	20	20	20	20	23	23		32	32	20	20
Study population	HFrEF	HFrEF	HFrEF	HFrEF	HFmrEF/HFpEF	HFmrEF/HFpEF		HF with type 2 diabetes	HF with type 2 diabetes	HFmrEF/HFpEF	HFmrEF/HFpEF
No. of participants	2373	2371	1863	1867	2997	2991		608	614	3131	3132
Age, mean, y	66.2 (11.0)	66.5 (10.8)	67.2 (10.8)	66.5 (11.2)	71.8 (9.3)	71.9 (9.6)		69 (63-76)^a^	70 (64-76)^a^	71.8 (9.6)	71.5 (9.5)
Sex, No. (%)											
Female	564 (23.8)	545 (23.0)	437 (23.5)	456 (24.4)	1338 (44.6)	1338 (44.7)		198 (32.6)	214 (34.9)	1364 (43.6)	1383 (44.2)
Male	1809 (76.2)	1826 (77.0)	1426 (76.5)	1411 (75.6)	1659 (55.4)	1653 (55.3)		410 (67.4)	400 (65.1)	1767 (56.4)	1749 (55.8)
Diabetes, No. (%)	993 (41.8)	990 (41.8)	927 (49.8)	929 (49.8)	1466 (48.9)	1472 (49.2)		608 (100)	614 (100)	1401 (44.7)	1405 (44.9)
Atrial fibrillation, No. (%)	916 (38.6)	902 (38.0)	664 (35.6)	705 (37.8)	1543 (51.5)	1514 (50.6)		576/1222 (47.1)^b^	576/1222 (47.1)^b^	1758 (56.1)	1794 (57.3)
Cause of heart failure, No. (%)											
Ischemic	1316 (55.5)	1358 (57.3)	983 (52.8)	946 (50.7)	1079 (36.0)	1038 (34.7)		712/1222 (58.3)^b^	712/1222 (58.3)^b^	NA	NA
Nonischemic	857 (36.1)	830 (35.0)	880 (47.2)	921 (49.3)	1917 (64.0)	1953 (65.3)		503/1222 (41.2)^b^	503/1222 (41.2)^b^	NA	NA
NYHA, No. (%)											
I/II	1606 (67.7)	1597 (67.4)	1399 (75.1)	1401 (75.0)	2432 (81.1)	2451 (81.9)		552/1222 (45.2)^b^	552/1222 (45.2)^b^	2314 (73.9)	2399 (76.6)
III/IV	767 (32.3)	774 (32.6)	464 (24.9)	466 (25.0)	562 (18.8)	539 (18.0)		614/1222 (50.2)^b^	614/1222 (50.2)^b^	817 (26.1)	732 (23.4)
LVEF, mean (SD), %	31.2 (6.7)	30.9 (6.9)	27.7 (6.0)	27.2 (6.1)	54.3 (8.8)	54.3 (8.8)		35 (28-47)^a^	35 (28-45)^a^	54.0 (8.6)	54.3 (8.9)
Median NT-proBNP (IQR), pg/mL	1428 (857-2655)	1446 (857-2641)	1887 (1077-3429)	1926 (1153-3525)	994 (501-1740)	946 (498-1725)		1817 (855-3659)	1741 (843-3582)	1011 (623-1751)^b^	1011 (623-1751)^b^
eGFR, mean (SD), mL/min/1.73 m^2^	66.0 (19.6)	65.5 (19.3)	61.8 (21.7)	62.2 (21.5)	60.6 (19.8)	60.6 (19.9)		49.2 (39.5-61.2)^a^	50.5 (40.5-64.6)^a^	61 (19)	61 (19)
Heart failure medication, No. (%)											
ARNI/ACEI/ARB	2257 (95.1)	2219 (93.6)	1654 (88.8)	1673 (89.6)	2493 (83.2)	2473 (82.7)		592 (97.4)	614 (100)	2442 (78.0)	2426 (77.5)
β-blocker	2278 (96.0)	2280 (96.2)	1765 (94.7)	1768 (94.7)	2598 (86.7)	2569 (85.9)		564 (92.8)	561 (91.4)	2592 (82.8)	2585 (82.5)
Mineralocorticoid receptor antagonist	1696 (71.5)	1674 (70.6)	1306 (70.1)	1355 (72.6)	1119 (37.3)	1125 (37.6)		403 (66.3)	385 (62.7)	1340 (42.8)	1327 (42.4)
Median follow-up, mo	18.2	18.2	16.0	16.0	26.2		26.2	9.2	9.2	27.6	27.6
ARR, %^c^	4.82	[Reference]	2.97	[Reference]	5.47		[Reference]	10.03	[Reference]	3.1	[Reference]
HR (95% CI)	0.74 (0.65-0.85)	1 [Reference]	0.75 (0.65-0.86)	1 [Reference]	0.79 (0.69-0.90)		1 [Reference]	0.71 (0.56-0.89)^d^	1 [Reference]	0.82 (0.73-0.92)	1 [Reference]

Abbreviations: ACEI, angiotensin-converting enzyme inhibitors; ARB, angiotensin receptor blocker; ARNI, angiotensin receptor neprilysin inhibitor; ARR, absolute relative risk; eGFR, estimated glomerular filtration rate; HF, heart failure; HFmrEF, heart failure with mildly reduced ejection fraction; HFpEF, heart failure with preserved ejection fraction; HFrEF, heart failure with reduced ejection fraction; HR, hazard ratio; LVEF, left ventricular ejection fraction; NT-proBNP, N-terminal pro-B-type natriuretic peptide; NYHA, New York Heart Association.

SI conversion factor: To convert NT-proBNP to nanograms per liter, multiply by 1.

^a^ Value shown as median (IQR).

^b^ Data by the group was not available.

^c^ ARR was derived from the reconstructed data.

^d^ HR for the time to the first occurrence of cardiovascular death and hospitalization for HF was used to match the objective of our analysis.

**Figure 1.**
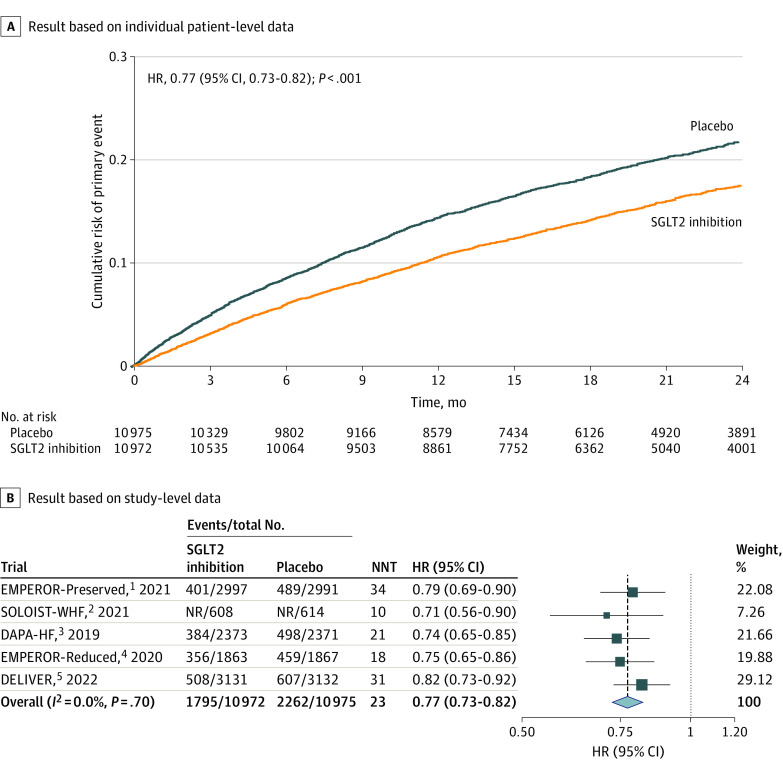
Cumulative Risk and Hazard Ratio (HR) of Primary Outcome for Sodium-Glucose Cotransporter 2 (SGLT2) Inhibitors vs Placebo NNT indicates number needed to treat.

The KM curve of pooled trial data indicated a consistently lower cumulative incidence of the primary outcome in the SGLT2 inhibitors vs placebo treatment group (HR, 0.77 [95% CI, 0.73-0.82]; *P* < .001) (Figure 1A). This was confirmed by the meta-analysis at the study level (HR, 0.77 [95% CI, 0.73-0.82]) (Figure 1B). Further analyses also showed a similar association of SGLT2 inhibitors with HF hospitalization (eFigure 7 in Supplement 1) and CV death (eFigure 8 in Supplement 1), but not statistically significant on all-cause mortality (eFigure 9 in Supplement 1).

**Figure 2.**
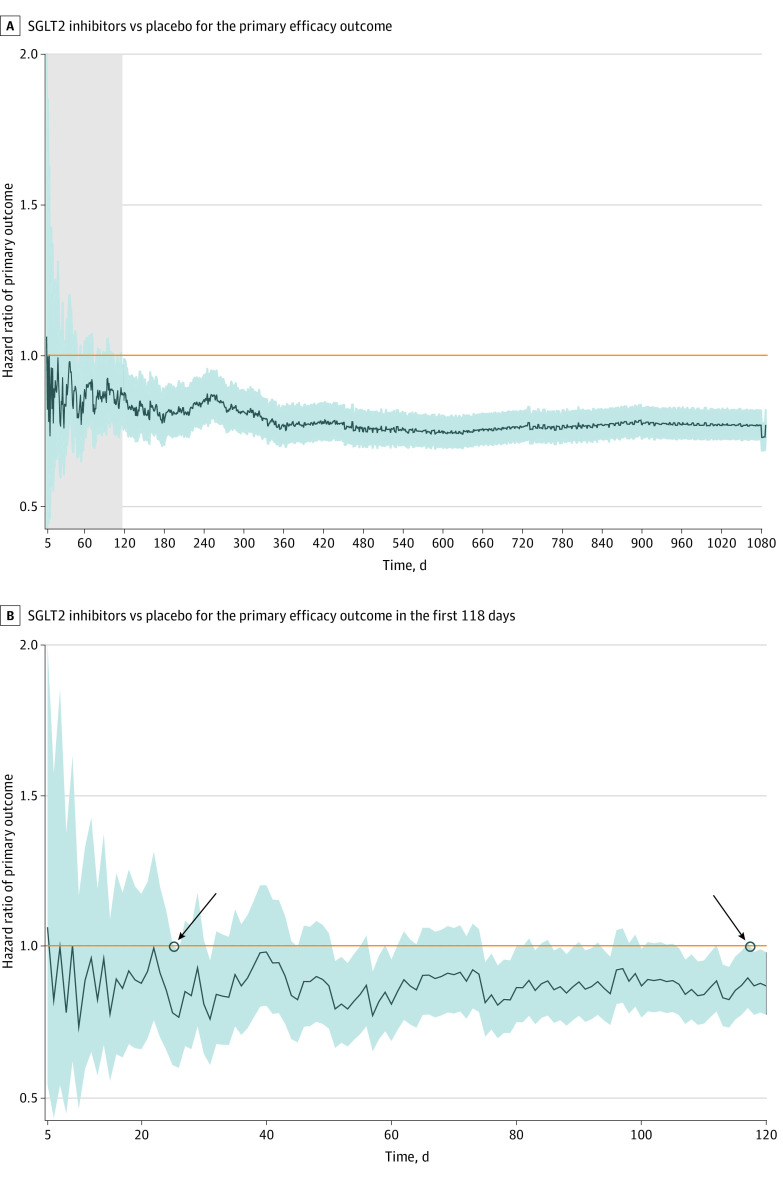
Time to First or Consistently Reach Statistically Significant Clinical Benefit Shaded regions indicate 95% CIs. In panel B, left arrow indicates the time to first nominal statistically significant clinical benefit (26 days [0.86 months]); right arrow, time to consistently reach statistically significant clinical benefit and sustain it thereafter (118 days [3.93 months]). SGLT2 indicates sodium-glucose cotransporter 2.

In line with Figure 1A, Figure 2 shows a reduction in the risk of the primary outcome from SGLT2 inhibitors over time. The benefit (HR <1.00) first reached statistical significance at 26 days (0.86 months) after randomization, and statistical significance was sustained from day 118 (3.93 months) onwards. eTable 3 in Supplement 1 also shows the time at which significance was reached for HF hospitalization (1.30 months; HR, 0.68 [95% CI, 0.52-0.98]) or CV death (19.03 months; HR, 0.88 [95% CI, 0.80-0.98]).

Our further analyses to determine the TTB at different clinically meaningful thresholds indicated that 0.19 (95% CI, 0.12-0.35) months were needed to prevent 1 HF hospitalization or CV death per 500 patients with the SGLT2 inhibitors treatment (ARR, 0.002). When moving the ARR threshold to 0.005, TTB would be 0.66 (95% CI, 0.43-1.13) months; with ARR threshold at 0.01, TTB would be 1.74 (95% CI, 1.07-2.61) months; and with ARR threshold at 0.02, the TTB would be 4.96 (95% CI, 3.18-7.26) months (Table 2). The estimates did not materially change after excluding SOLOIST-WHF^2^ only with individuals after a worsening HF episode. The TTB to specific ARR thresholds varied across different subgroups on the primary outcome. In general, the mean TTB was lower in patients with diabetes (3.68 [95% CI, 2.18-6.78] vs 5.70 [95% CI, 3.20-13.41] at ARR threshold of 0.02) or HFrEF (95% CI, 3.39 [2.01-6.42] vs 7.22 [95% CI, 4.07-79.07]) at ARR threshold of 0.02 (Table 3). Additional analysis indicated that the TTB was shorter for HF hospitalization than for CV death (eTable 4 in Supplement 1).

**Table 2.** Time to Benefit at Specific Thresholds of Absolute Risk Reduction^a^

Absolute risk reduction threshold	Time to benefit, mean (95% CI), mo				
	EMPEROR-Preserved,^1^ 2021	Add EMPEROR-Reduced,^4^ 2020	Add DAPA-HF,^3^ 2019	Add SOLOIST-WHF,^2^ 2021	Add DELIVER,^5^ 2022
0.002	0.23 (0.12-0.72)	0.17 (0.09-0.45)	0.20 (0.12-0.41)	0.19 (0.11-0.41)	0.19 (0.12-0.35)
0.005	0.84 (0.42-3.17)	0.61 (0.34-1.42)	0.65 (0.40-1.26)	0.63 (0.39-1.21)	0.66 (0.43-1.13)
0.010	2.36 (1.14-59.13)	1.68 (0.94-3.64)	1.70 (1.06-3.08)	1.62 (1.02-2.84)	1.74 (1.07-2.61)
0.020	7.85 (3.35-194.29)	5.05 (2.88-11.08)	4.81 (3.06-8.29)	4.38 (2.89-7.11)	4.96 (3.18-7.26)

^a^ Each study is added in succession starting from left to right, and the time to benefit is reestimated with the far-right column being the summary time to benefit after including all studies.

**Table 3.** Subgroup Analysis for Time to Benefit at Specific Thresholds of Absolute Risk Reduction by Different Characteristics

Study characteristics	Time to benefit, mean (95% CI), mo			
	Absolute risk reduction threshold			
	0.002	0.005	0.010	0.020
Individual trials				
EMPEROR-Preserved,^1^ 2021 (n = 5988)	0.23 (0.12-0.72)	0.84 (0.42-3.17)	2.36 (1.14-59.13)	7.85 (3.35-194.29)
EMPEROR-Reduced,^4^ 2020 (n = 3730)	0.09 (0.04-0.49)	0.32 (0.14-1.64)	0.85 (0.38-4.26)	2.45 (1.08-12.05)
DAPA-HF,^3^ 2019 (n = 4744)	0.23 (0.11-0.95)	0.71 (0.35-2.72)	1.70 (0.85-6.36)	4.33 (2.14-16.17)
SOLOIST-WHF,^2^ 2021 (n = 1222)	0.06 (0.01-1.19)	0.17 (0.04-2.10)	0.40 (0.10-3.18)	0.94 (0.28-4.95)
DELIVER,^5^ 2022 (n = 6263)	0.19 (0.10-0.58)	0.70 (0.35-2.51)	2.00 (0.98-14.24)	6.69 (2.90-197.93)
SGLT2 inhibitors				
Dapagliflozin (n = 11 007)	0.21 (0.12-0.50)	0.72 (0.41-1.55)	1.90 (1.11-3.85)	5.53 (3.27-11.30)
Empagliflozin (n = 9718)	0.17 (0.10-0.45)	0.61 (0.34-1.42)	1.68 (0.94-3.64)	5.05 (2.88-11.08)
Sotagliflozin (n = 1222)	0.06 (0.01-1.19)	0.17 (0.04-2.10)	0.40 (0.10-3.18)	0.94 (0.28-4.95)
Type 2 diabetes				
Yes (n = 8155)	0.20 (0.10-0.63)	0.61 (0.32-1.54)	1.47 (0.82-3.10)	3.68 (2.18-6.78)
No (n = 7529)	0.25 (0.13-0.66)	0.80 (0.44-1.91)	2.03 (1.14-4.52)	5.70 (3.20-13.41)
LVEF				
HF mildly reduced/preserved (n = 12 251)	0.21 (0.12-0.46)	0.76 (0.44-1.61)	2.17 (1.27-4.54)	7.22 (4.07-79.07)
HF reduced (n = 8474)	0.16 (0.08 0.42)	0.50 (0.28-1.18)	1.27 (0.73-2.64)	3.39 (2.01-6.42)

Abbreviations: HF, heart failure; LVEF, left ventricular ejection fraction; SGLT2, sodium-glucose cotransporter 2.

## Discussion

In this pooled analysis of more than 21 000 individual patient data from 5 RCTs, we found a clinical benefit of SGLT2 inhibitors associated with reduced CV death or HF hospitalization and found that the benefit started within 1 month and sustained from approximately 4 months onwards. Further analyses indicated that TTB to prevent 1 clinical event for 500, 200, 100, and 50 patients with HF receiving SGLT2 inhibitors was 0.19, 0.66, 1.74 and 4.96 months, respectively, suggesting the early benefit of SGLT2 inhibitors among patients with HF. It is noteworthy to mention that patients may obtain quicker treatment benefits on the risk of HF hospitalization, or among patients with diabetes or reduced ejection fraction. Our study underscored the urgency of initiating SGLT2 inhibitor use to overcome clinical inertia in patients with chronic HF.

Current established therapies such as angiotensin receptor-neprilysin inhibitors (ARNI), angiotensin-converting enzyme inhibitors (ACEI), angiotensin receptor blockers, and β-blockers have been proven to reduce hospitalizations and mortality risks in patients with HFrEF.^23,24^ The SGLT2 inhibitor is a novel class of antidiabetic drugs and several CV outcomes trials have shown its cardiorenal benefits in patients with type 2 diabetes.^25,26,27^ Recent trials or systematic reviews have found that SGLT2 inhibitors could prevent CV deaths and HF hospitalizations among patients with HFpEF or HFrEF, and treatment effects were consistent across various individual characteristics.^1,2,3,4,20,28,29^ As a result of the growing body of evidence, McMurray et al^9^ proposed a new algorithm for the sequencing of foundational treatments which was simultaneous initiation with a β-blocker and an SGLT2 inhibitor. However, uncertainties still existed for the current HF treatment.^6,10^ Currently, the use of comprehensive medical therapies remains suboptimal in clinical practice. Awareness of the timing of treatment benefits to clinicians and patients, especially for this new drug class of SGLT2 inhibitor, may be critical to promote faster and more widespread adoption of those highly efficacious therapies.

Recently, TTB has been increasingly discussed to understand the benefits and harms of treatment to an individual patient. However, to our knowledge, few clinical trials reported such information. Previously, TTB was estimated by visually identifying the time point at which the curves separate.^11,12^ This approach is subject to visual bias.

In our study, we first assessed the TTB by estimating the timing until the treatment effect first or consistently reached statistical significance based on a *P* < .05, which was also adopted in prior analyses in DELIVER,^30^ DAPA-HF,^31^ and SOLOIST-WHF.^32^ Similarly, our result suggested early and sustained clinical benefits from SGLT2 inhibitors in the range of 1 month. Meanwhile, to avoid the estimation heavily relying on this arbitrary *P* value, we also adopted the method proposed by Lee et al^14,16^ to calculate the time to reach the clinically meaningful ARR. We identified that the clinical benefit of SGLT2 inhibitors first reached statistical significance within 1 month after randomization and was sustained from 4 months onwards. Furthermore, we found that it only took approximately 5 months on average to prevent 1 composite event in 50 patients, suggesting that for most patients with a life expectancy greater than 5 months, the benefits of SGLT2 inhibitors may likely outweigh their harms. Of note, it seemed that patients with HFrEF or diabetes may benefit from the treatment of SGLT2 inhibitors more rapidly than their counterparts. Taken together with the recent findings of empagliflozin in patients hospitalized for acute HF^33^ and other evidence including the early initiation and continuation among survivors of acute myocardial infarction,^28,31,34^ we believed that early treatment of SGLT2 inhibitors may be effective for most populations with HF and any delay in therapy exposed patients to substantial excess risk.

### Strengths and Limitations

To our knowledge, this study was the first to quantitatively estimate the TTB at various absolute benefit thresholds for SGLT2 inhibitors among patients with HF. These results could help the clinician better optimize HF drug treatments and fill the evidence gap among the current HF guidelines.

However, several limitations of this study deserve mention. First, our study was a post hoc analysis of the patients-level efficacy but not safety data, which prevented us from further assessing the time to harm (such as the genital infections from SGLT2 inhibitors). Although the rate of adverse events is similar,^28,29^ awareness of this information may change clinical management decisions based on values and preferences of the individual. Second, although trials in our study had a similar design, these reconstructed data did not include covariates reflecting heterogeneous characteristics and different clinical scenarios, which allowed us to perform further subgroup analyses, for example, patients with New York Heart Association I/II vs III/IV. Third, we systematically searched the publications and endeavored to include all the completed RCTs. However, the limited number of included studies did not facilitate TTB estimations on all-cause death and left uncertainties over the drug-specific TTB estimations, such as sotagliflozin. Fourth, like many clinical trials, our estimation on TTB was to show the early benefit at the population level and may not apply to individual patients, who may need the clinician’s individualized assessment. Additionally, the absence of head-to-head comparisons between SGLT2 inhibitors and other established therapies (eg, β-blockers, ACEI, ARNI, and mineralocorticoid receptor antagonist) precluded a more complete report on TTB estimations or the order of drug initiation.

## Conclusion

This comparative effectiveness research study found that most patients with HF (life expectancy greater than 5 months) could benefit from the treatment of SGLT2 inhibitors. These findings suggest support for the decision to initiate SGLT2 inhibitors early for patients with HF, particularly for those with diabetes or HFrEF.

## Notes

Supplement 1. **eAppendix.** Search strategy of PubMed for RCT **eFigure 1.** Flowchart of the Search, Selection, and Inclusion of the Studies **eFigure 2.** Reconstruct KM Curve and Original KM Curve (DAPA-HF) **eFigure 3.** Reconstruct KM Curve and Original KM Curve (EMPEROR-Reduced) **eFigure 4.** Reconstruct KM Curve and Original KM Curve (EMPEROR-Preserved) **eFigure 5.** Reconstruct KM Curve and Original KM Curve (SOLOIST-WHF) **eFigure 6.** Reconstruct KM Curve and Original KM Curve (DELIVER) **eFigure 7.** Cumulative Risk and Hazard Ratio of First HF Hospitalization for SGLT2 Inhibitors vs Placebo **eFigure 8.** Cumulative Risk and Hazard Ratio of CV Death for SGLT2 Inhibitors vs Placebo **eFigure 9.** Cumulative Risk and Hazard Ratio of All-Cause Mortality for SGLT2 Inhibitors vs Placebo **eTable 1.** Definition for the Primary Outcome for Each Include Trials **eTable 2.** Risk of Bias Assessment of Included Trials **eTable 3.** Time to Consistently Reach the Statistically Significant Clinical Benefit **eTable 4.** Time to Benefit (Months) at Specific Thresholds of Absolute Risk Reduction

Supplement 2.
Data Sharing Statement
