# Supplementary material for: Time to Benefit of Sodium-Glucose Cotransporter-2 Inhibitors Among Patients With Heart Failure
Source: JAMA Netw Open. 2023 Aug 24;6(8):e2330754. doi: 10.1001/jamanetworkopen.2023.30754 (PMC10450563; doi:10.1001/jamanetworkopen.2023.30754)
Supplement: Supplement 1. — eAppendix. Search strategy of PubMed for RCT eFigure 1. Flowchart of the Search, Selection, and Inclusion of the Studies eFigure 2. Reconstruct KM Curve and Original KM Curve (DAPA-HF) eFigure 3. Reconstruct KM Curve and Original KM Curve (EMPEROR-Reduced) eFigure 4. Reconstruct KM Curve and Original KM Curve (EMPEROR-Preserved) eFigure 5. Reconstruct KM Curve and Original KM Curve (SOLOIST-WHF) eFigure 6. Reconstruct KM Curve and Original KM Curve (DELIVER) eFigure 7. Cumulative Risk and Hazard Ratio of First HF Hospitalization for SGLT2 Inhibitors vs Placebo eFigure 8. Cumulative Risk and Hazard Ratio of CV Death for SGLT2 Inhibitors vs Placebo eFigure 9. Cumulative Risk and Hazard Ratio of All-Cause Mortality for SGLT2 Inhibitors vs Placebo eTable 1. Definition for the Primary Outcome for Each Include Trials eTable 2. Risk of Bias Assessment of Included Trials eTable 3. Time to Consistently Reach the Statistically Significant Clinical Benefit eTable 4. Time to Benefit (Months) at Specific Thresholds of Absolute Risk Reduction [file jamanetwopen-e2330754-s001.pdf]

Supplemental Online Content

Chen K, Nie Z, Shi R, et al. Time to benefit of sodium-glucose cotransporter-2 inhibitors among patients with heart failure. *JAMA Netw Open.* 2023;6(8):e2330754. doi:10.1001/jamanetworkopen.2023.30754

eAppendix. Search Strategy of PubMed for RCT

eFigure 1. Flowchart of the Search, Selection, and Inclusion of the Studies

eFigure 2. Reconstruct KM Curve and Original KM Curve (DAPA-HF)

eFigure 3. Reconstruct KM Curve and Original KM Curve (EMPEROR-Reduced)

eFigure 4. Reconstruct KM Curve and Original KM Curve (EMPEROR-Preserved)

eFigure 5. Reconstruct KM Curve and Original KM Curve (SOLOIST-WHF)

eFigure 6. Reconstruct KM Curve and Original KM Curve (DELIVER)

eFigure 7. Cumulative Risk and Hazard Ratio of First HF Hospitalization for SGLT2 Inhibitors vs Placebo

eFigure 8. Cumulative Risk and Hazard Ratio of CV Death for SGLT2 Inhibitors vs Placebo

eFigure 9. Cumulative Risk and Hazard Ratio of All-Cause Mortality for SGLT2 Inhibitors vs Placebo

eTable 1. Definition for the Primary Outcome for Each Include Trials

eTable 2. Risk of Bias Assessment of Included Trials

eTable 3. Time to Consistently Reach the Statistically Significant Clinical Benefit

eTable 4. Time to Benefit (Months) at Specific Thresholds of Absolute Risk Reduction

This supplemental material has been provided by the authors to give readers additional information about their work.

eAppendix. **Search Strategy of PubMed for RCT**

((("SGLT2"[Mesh] OR "sodium glucose transporter 2"[Mesh] OR "dapagliflozin"[tiab] OR "canagliflozin"[tiab] OR "empagliflozin"[tiab] OR "sotagliflozin"[tiab] OR "ertugliflozin"[tiab] OR "ipragliflozin"[tiab] OR "luseogliflozin"[tiab] OR "tofogliflozin"[tiab])) AND (("randomized controlled trial"[pt] OR " controlled clinical trial "[pt] OR " randomized "[tiab] OR " placebo"[tiab] OR "drug therapy"[sh] OR "randomly"[tiab] OR "trial"[tiab] OR "groups"[tiab]))) AND (("Heart failure "[Mesh] OR "*Heart failure "[tiab] OR "*Cardiac Failure "[tiab] OR " congestive Cardiac Failure "[tiab] OR " congestive heart Failure "[tiab] OR " Heart Decompensation "[tiab] OR "Decompensation"[tiab]) OR " Right-Sided Heart Failure "[Mesh] OR " left-Sided Heart Failure "[tiab] OR " Myocardial Failure "[tiab] OR " Heart Failure with preserved Ejection Fraction "[tiab] OR " HFpEF "[Mesh] OR " Heart Failure with reduced Ejection Fraction "[tiab] OR " HFrEF "[tiab]))

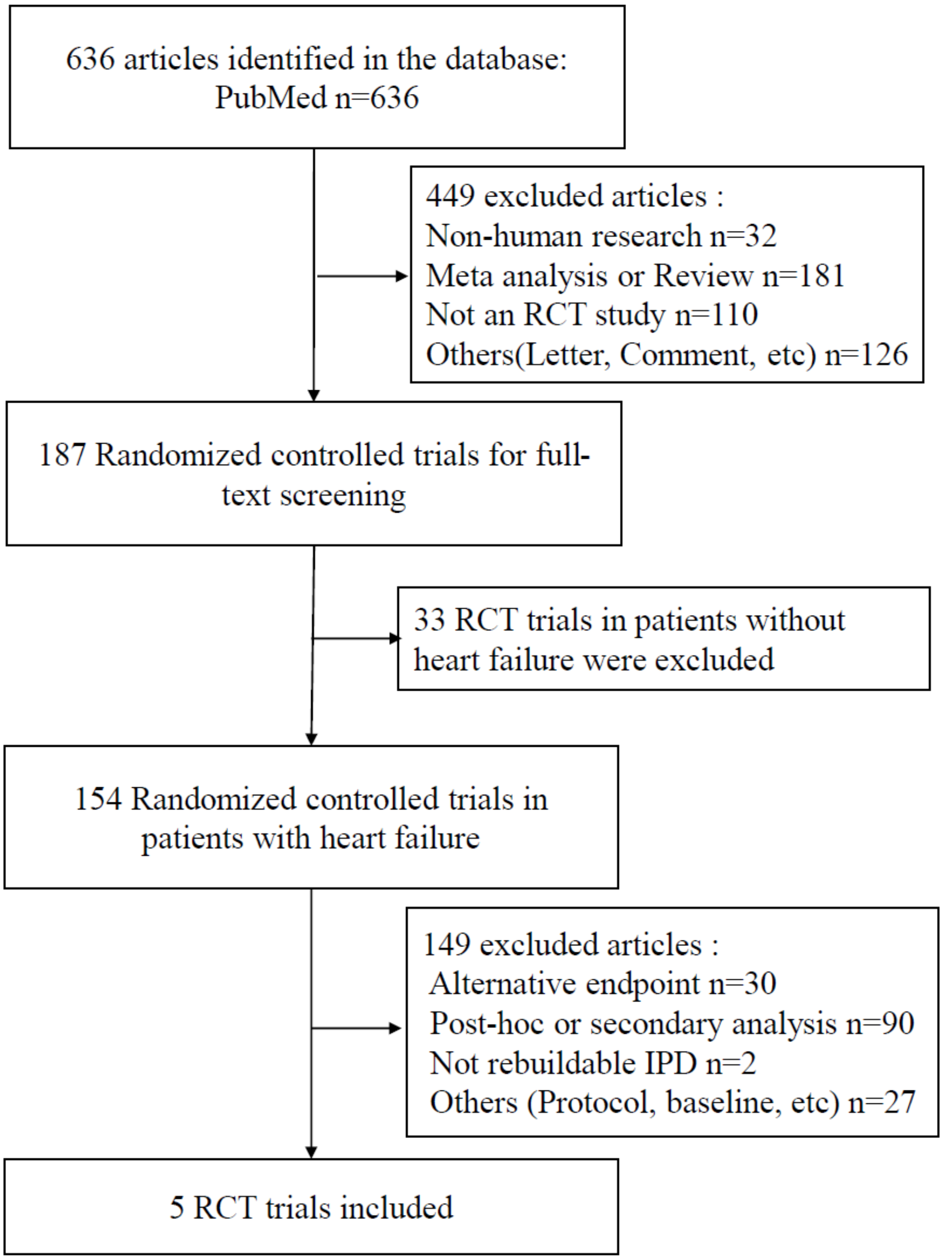

eFigure 1 Flow chart of the search, selection, and inclusion of the studies

© 2023 Chen K et al. *JAMA Network Open.*

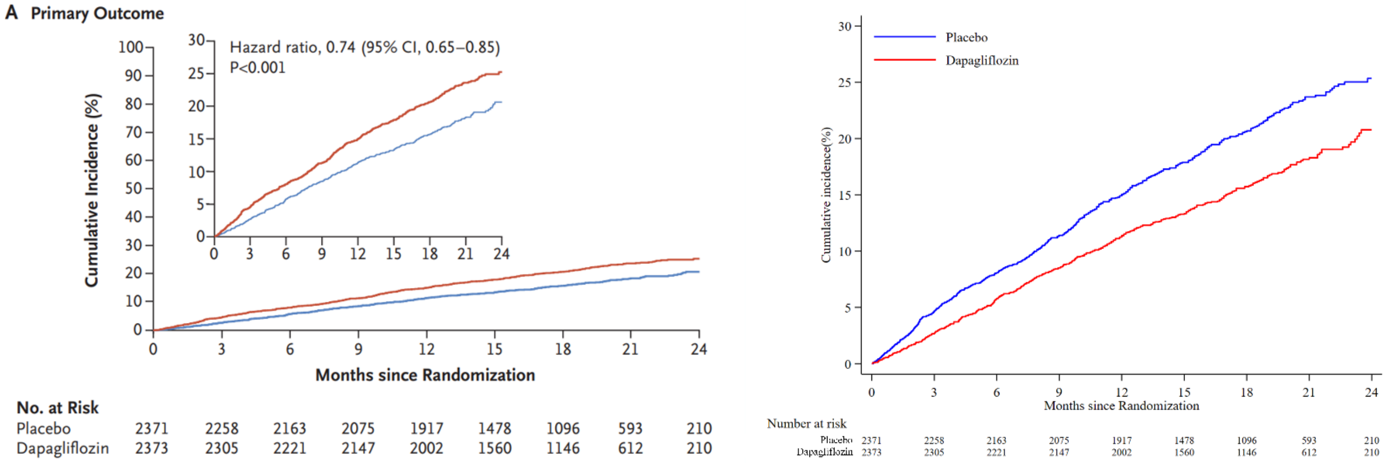

eFigure 2 Reconstruct KM curve and original KM curve (DAPA-HF)

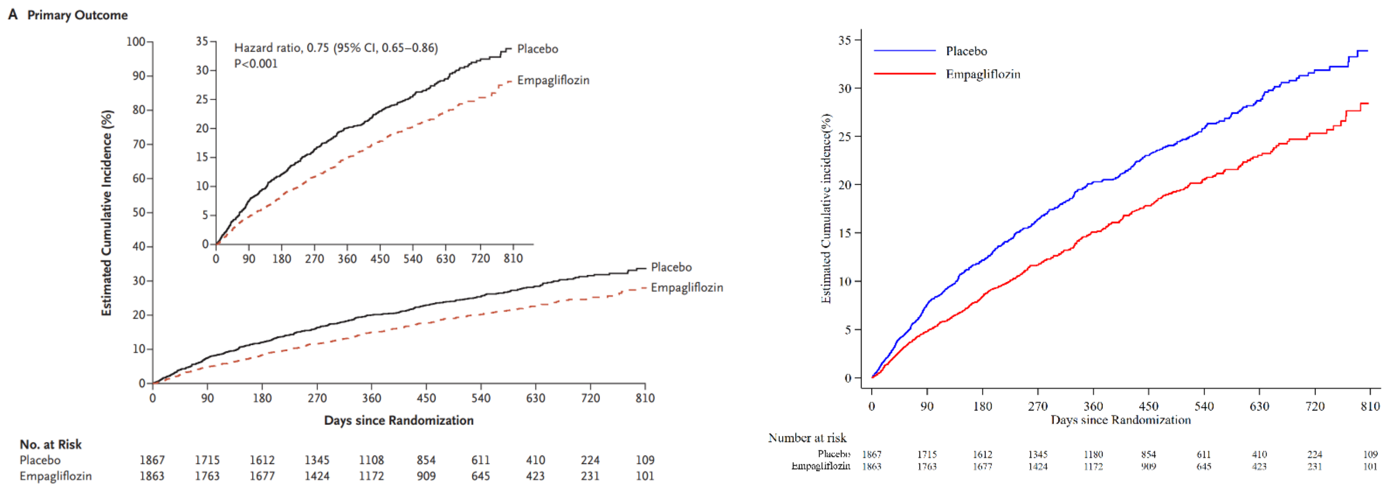

eFigure 3 Reconstruct KM curve and original KM curve (EMPEROR-reduced)

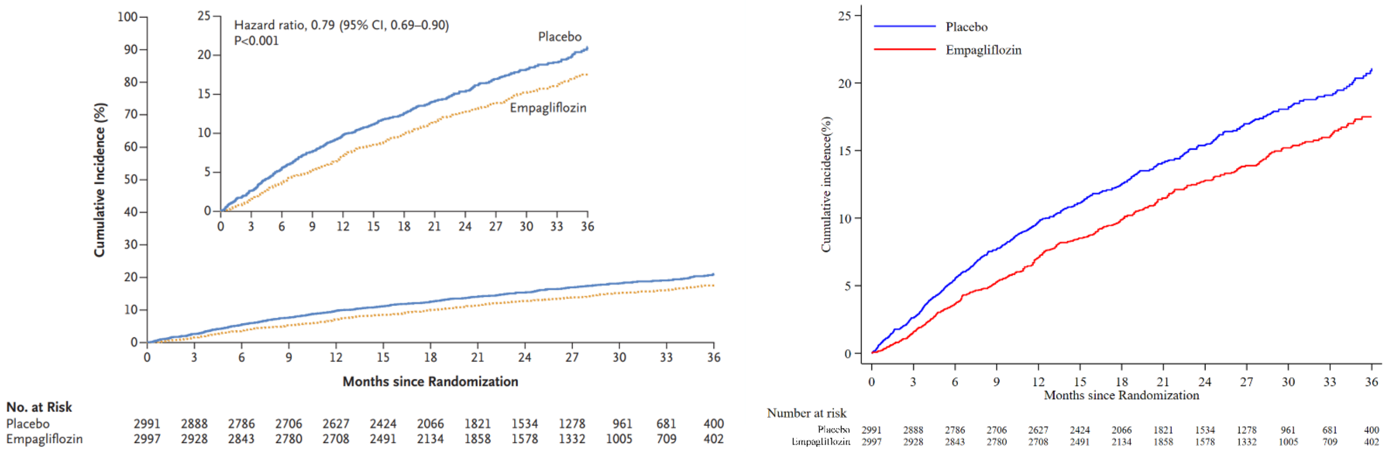

eFigure 4 Reconstruct KM curve and original KM curve (EMPEROR-Preserved)

© 2023 Chen K et al. *JAMA Network Open.*

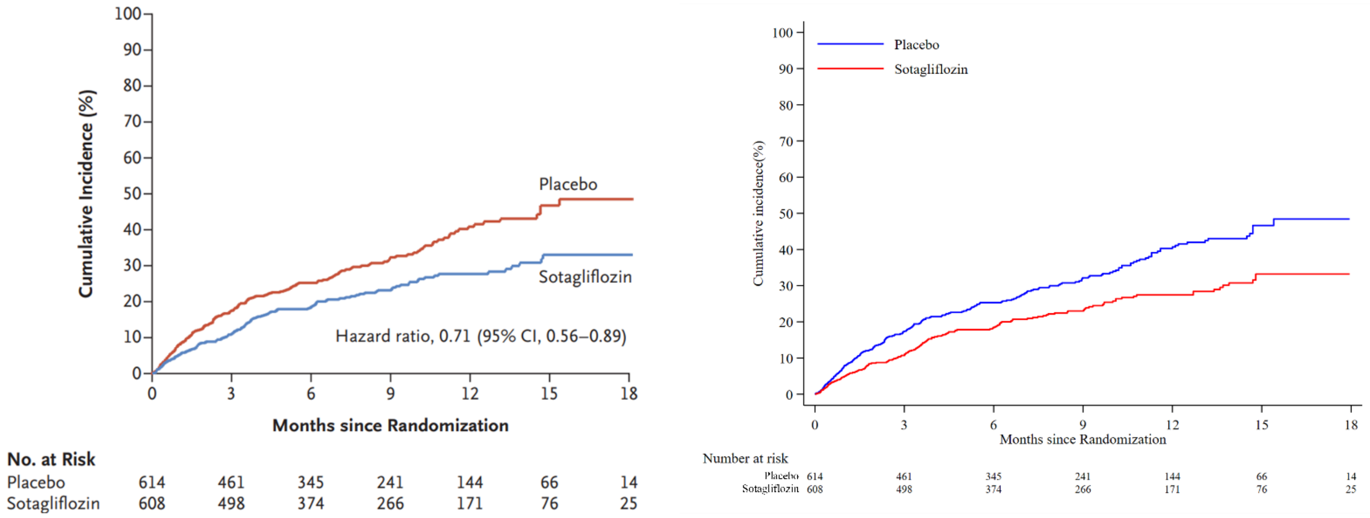

eFigure 5 Reconstruct KM curve and original KM curve (SOLOIST-WHF)

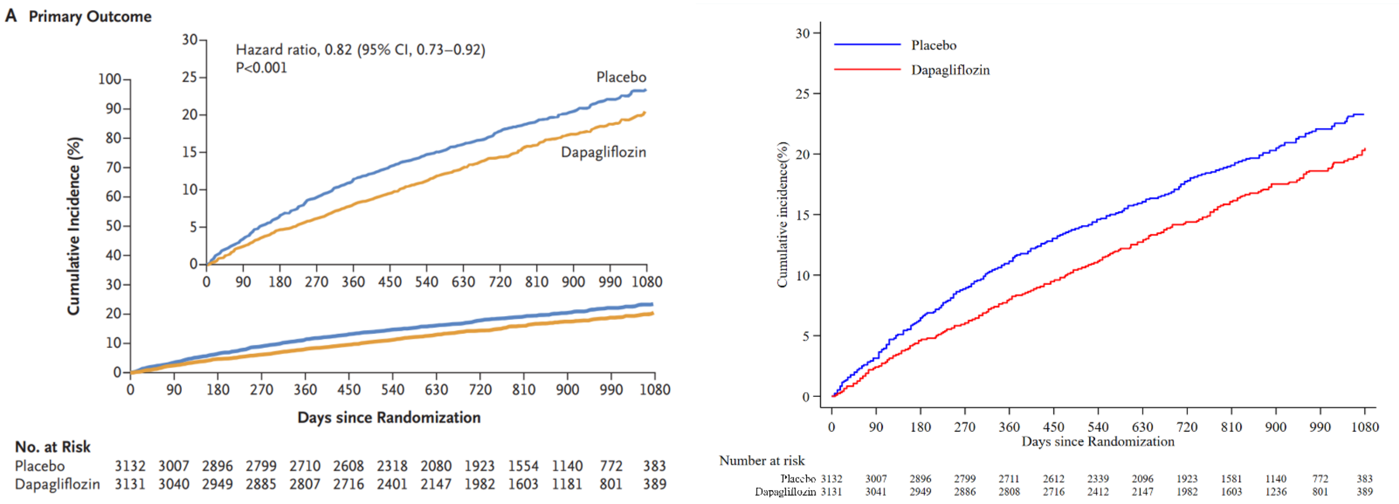

eFigure 6 Reconstruct KM curve and original KM curve (DELIVER)

© 2023 Chen K et al. *JAMA Network Open.*

A

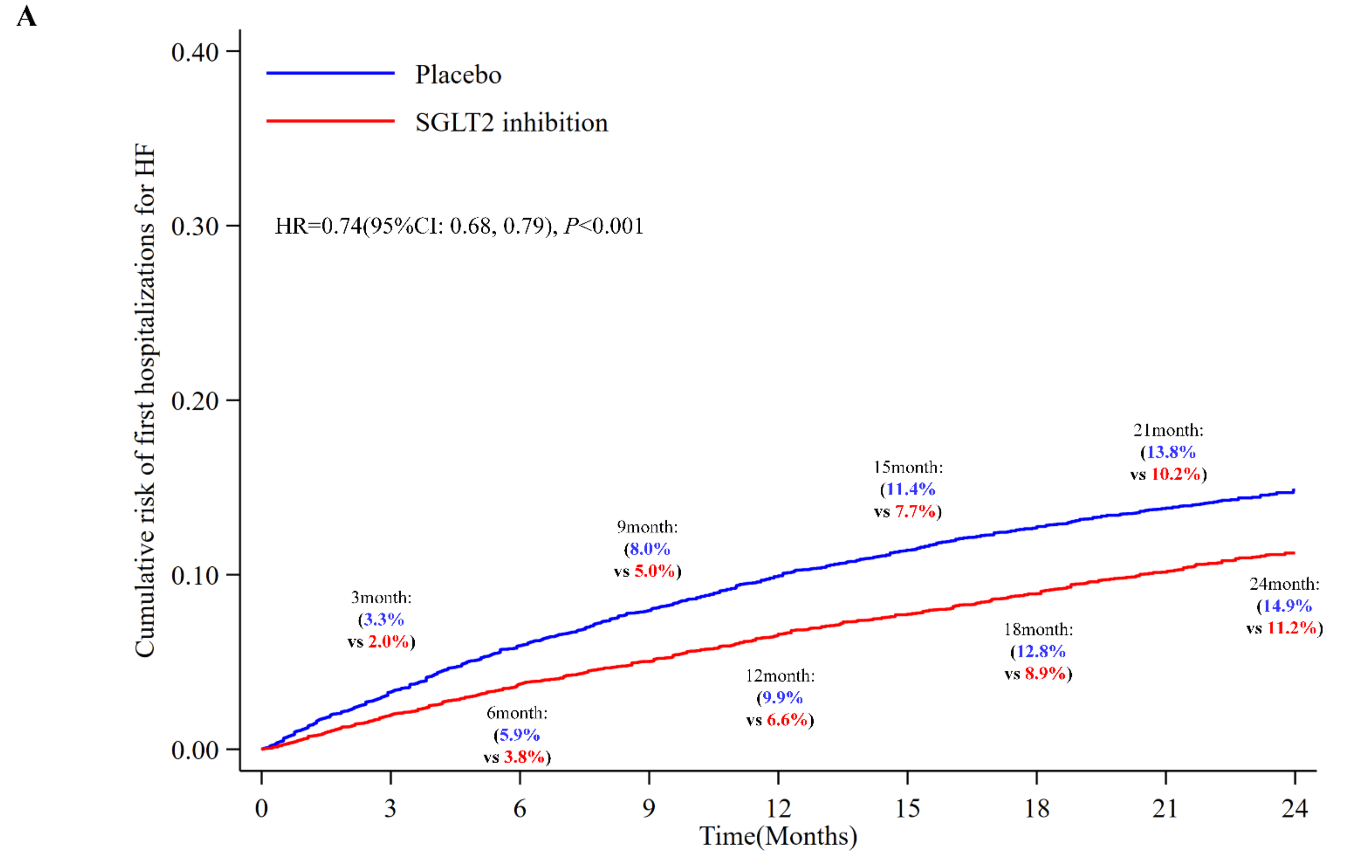

Number at risk

	0	3	6	9	12	15	18	21	24
Placebo	10367	9918	9521	9006	8450	7441	6186	4962	3952
SGLT2 inhibition	10358	10000	9639	9168	8616	7612	6259	5004	3957

B. First hospitalization for HF

	Event/N SGLT2 inhibition	Event/N Placebo	NNT	HR(95%CI)	%Weight
EMPEROR-Preserved	240/2991	343/2997	29	0.710(0.604, 0.835)	23.11
DAPA-HF	231/2373	318/2371	27	0.700(0.590, 0.830)	20.89
EMPEROR-Reduced	232/1863	324/1867	20	0.690(0.589, 0.808)	24.23
DELIVER	364/3131	453/3132	35	0.790(0.688, 0.907)	31.77
Overall (I^2=0.0%, *P*=0.557)	1067/10358	1438/10367	28	**0.727(0.673, 0.786)**	100

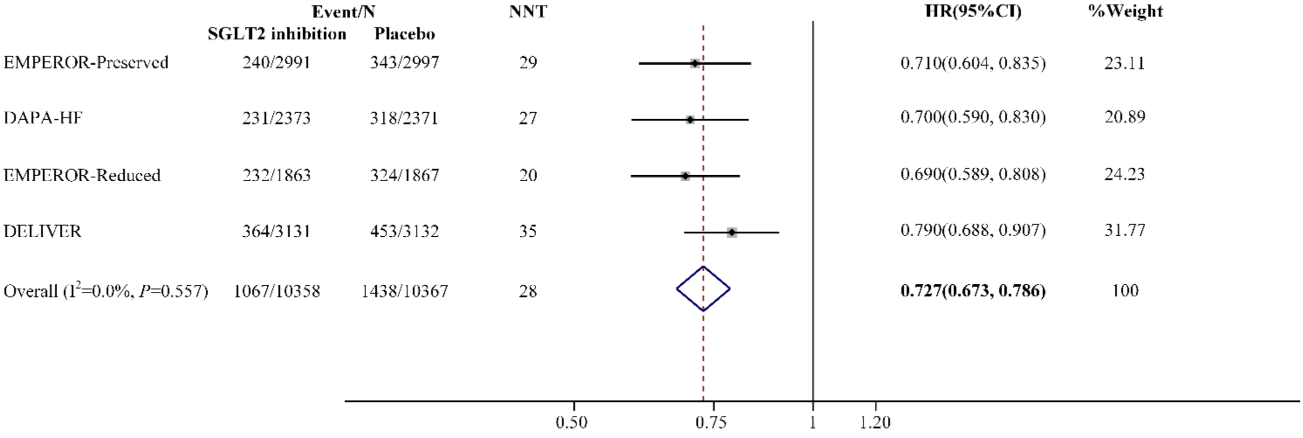

eFigure 7 Cumulative risk and hazard ratio of first HF hospitalization for SGLT2 inhibitors vs placebo

© 2023 Chen K et al. *JAMA Network Open.*

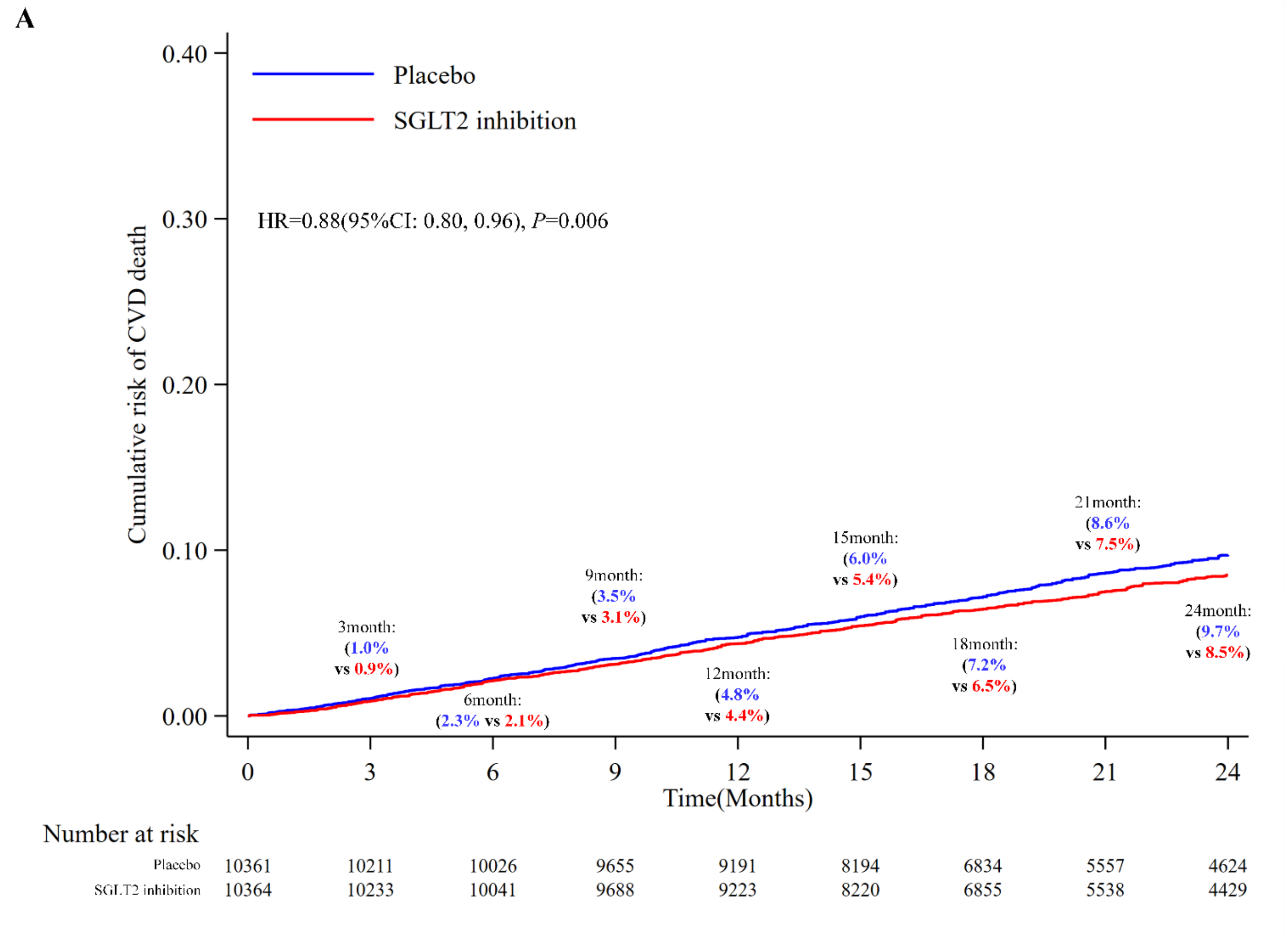

B. CVD Death

	Event/N		NNT	HR(95%CI)	%Weight
	SGLT2 inhibition	Placebo			
EMPEROR-Preserved	209/2997	230/2991	139	0.910(0.760, 1.090)	25.46
DAPA-HF	229/2373	276/2371	50	0.820(0.688, 0.977)	26.90
EMPEROR-Reduced	175/1863	192/1867	112	0.920(0.753, 1.124)	20.59
DELIVER	232/3131	262/3132	104	0.880(0.739, 1.048)	27.05
Overall (I^2=0.0%, *P*=0.815)	845/10364	960/10361	89	**0.879(0.802, 0.963)**	100

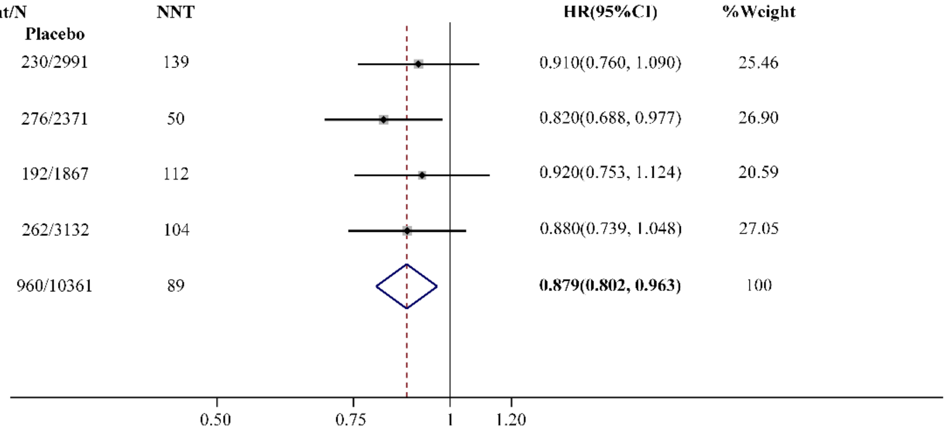

eFigure 8 Cumulative risk and hazard ratio of CVD death for SGLT2 inhibitors vs placebo

© 2023 Chen K et al. *JAMA Network Open.*

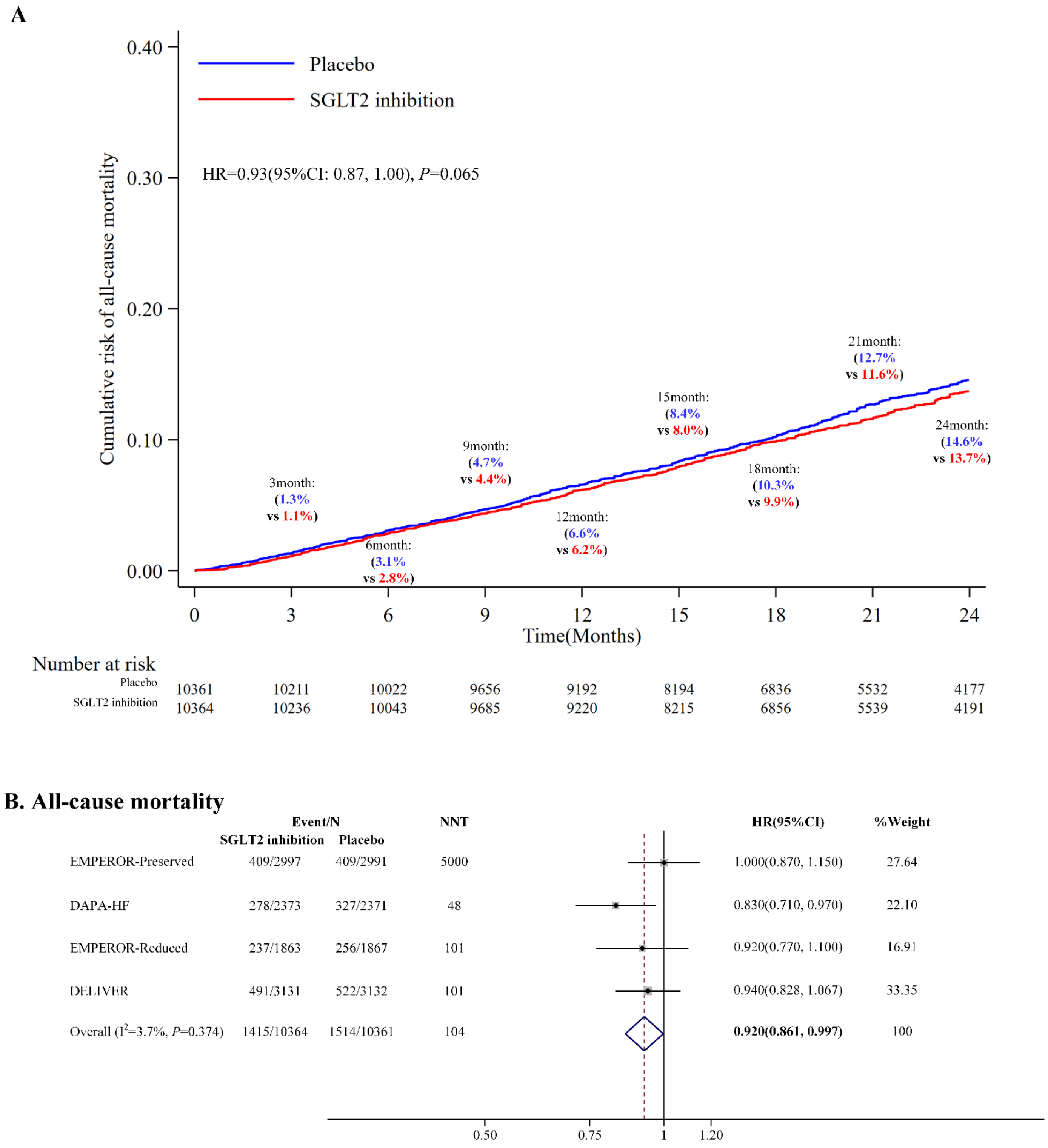

eFigure 9 Cumulative risk and hazard ratio of all-cause mortality for SGLT2 inhibitors vs placebo

© 2023 Chen K et al. *JAMA Network Open.*

eTable 1 Definition for the primary outcome for each include trials

Trial	Definition
DAPA-HF	The primary outcome was a composite of worsening heart failure or death from cardiovascular causes. An episode of worsening heart failure was either an unplanned hospitalization or an urgent visit resulting in intravenous therapy for heart failure.
EMPEROR-Reduced	The composite primary endpoint for this trial is the time to first event of adjudicated CV death or adjudicated HHF in patients with Heart Failure with reduced Ejection Fraction (HFrEF).
EMPEROR-Preserved	The primary outcome was a composite of adjudicated cardiovascular death or hospitalization for heart failure, analyzed as the time to the first event.<br>For further clarification: Adjudicated CV death always includes death adjudicated as death due to undetermined cause. This is applicable throughout all analyses wherever adjudicated CV death is mentioned.
SOLOIST-WHF	The trial was originally designed with a primary endpoint of the first occurrence of either death from cardiovascular causes or hospitalization for heart failure, as described in the trial protocol. But was changed the primary end point to the total number of deaths from cardiovascular causes and hospitalizations and urgent visits for heart failure (first and subsequent) in order to increase the power of the trial. Please note, in order to be consistent, we extract the original primary endpoint data.
DELIVER	The primary outcome was a composite of worsening heart failure, which was defined as either an unplanned hospitalization for heart failure or an urgent visit for heart failure, or cardiovascular death.

eTable 2. Risk of Bias Assessment of included trials

	EMPEROR-Preserved	EMPEROR-Reduced	DAPA-HF	SOLOIST-WHF	DELIVER
Random sequence generation	Low	Low	Low	Low	Low
Incomplete outcome data	Low	Low	Low	Low	Low
Selective reporting	Low	Low	Low	Low	Low
Allocation concealment	Low	Low	Low	Low	Low
Blinding of outcome assessment	Low	Low	Low	Low	Low
Overall	Low	Low	Low	Low	Low

© 2023 Chen K et al. *JAMA Network Open.*

eTable 3. Time to consistently reach statistically significant clinical benefit

	EMPEROR-Preserved	+EMPEROR-Reduced	+DAPA-HF	+SOLOIST-WHF[1]	+DELIVER
**Primary Outcome**					
HRs(95%CI)	0.83(0.69, 0.99)	0.84(0.72, 0.99)	0.85(0.73, 0.97)	0.85(0.75, 0.97)	0.87(0.77, 0.98)
Months	11.60	5.50	4.90	4.17	3.93
**HF Hospitalization**					
HRs(95%CI)	0.42(0.19, 0.91)	0.69(0.49, 0.99)	0.73(0.54, 0.99)	-	0.68(0.52, 0.98)
Months	0.83	1.40	1.37	-	1.30
**CVD Death**					
HRs(95%CI)	-	-	0.89(0.78, 0.99)	-	0.88(0.80, 0.98)
Months	-	-	18.77	-	19.03

[1]Data cannot be rebuilt due to unavailable KM curves for SOLOIST-WHF trial.

© 2023 Chen K et al. *JAMA Network Open.*

eTable 4. Time to benefit (months) at specific thresholds of absolute risk reduction

	EMPEROR-Preserved	+EMPEROR-Reduced	+DAPA-HF	+DELIVER
**Primary outcome**				
Threshold				
0.002	0.23(0.12, 0.72)	0.17(0.09, 0.45)	0.20(0.12, 0.41)	0.19(0.12, 0.35)
0.005	0.84(0.42, 3.17)	0.61(0.34, 1.42)	0.65(0.40, 1.26)	0.67(0.44, 1.15)
0.01	2.36(1.14, 59.13)	1.68(0.94, 3.64)	1.70(1.06, 3.08)	1.79(1.20, 2.96)
0.02	7.85(3.35, 194.29)	5.05(2.88, 11.08)	4.81(3.06, 8.29)	5.30(3.57, 8.61)
**HF Hospitalization**				
Threshold				
0.002	0.25(0.11, 1.07)	0.19(0.09, 0.57)	0.22(0.12, 0.51)	0.18(0.11, 0.32)
0.005	0.99(0.45, 4.49)	0.71(0.36, 1.80)	0.79(0.45, 1.62)	0.66(0.43, 1.15)
0.01	2.98(0.56, 6.61)	2.05(1.09, 4.42)	2.16(1.31, 3.96)	1.93(1.27, 3.20)
0.02	10.00(4.18, 298.23)	6.29(3.59, 12.61)	6.32(3.77, 9.93)	6.22(4.15, 10.16)
**CVD Death**				
Threshold				
0.002	6.34(1.41, 85.57)	3.59(1.04, 37.35)	3.08(1.11, 16.85)	4.52(1.69, 17.08)
0.005	18.14(3.85, 181.63)	11.58(3.25, 96.48)	8.29(3.30, 55.42)	10.94(4.93, 51.97)
0.01	45.90(8.37, 262.95)	297.94(8.78, 10106.82)	18.73(8.19, 137.36)	22.01(11.59, 143.50)
0.02	NA[1]	NA[1]	51.43(20.19, 207.20)	47.66(25.00, 206.08)

[1]The treatment effect of SGLT2 inhibition was not significant for CVD death in EMPEROR-Preserved and EMPEROR-Reduced trials, the time to benefit of CVD death was not estimable.

Data cannot be rebuilt due to unavailable KM curves for HF Hospitalization and CVD Death in SOLOIST-WHF trial.

© 2023 Chen K et al. *JAMA Network Open.*
