# Supplementary material for: Time to Benefit of Sodium-Glucose Cotransporter-2 Inhibitors Among Patients With Heart Failure
Source: JAMA Netw Open. 2023 Aug 24;6(8):e2330754. doi: 10.1001/jamanetworkopen.2023.30754 (PMC10450563; doi:10.1001/jamanetworkopen.2023.30754)
Supplement: Supplement 2. — Data Sharing Statement [file jamanetwopen-e2330754-s002.pdf]

Data Sharing Statement

Chen. Time to Benefit of Sodium-Glucose Cotransporter-2 Inhibitors Among Patients With Heart Failure. *JAMA Netw Open*. Published August 24, 2023.
doi:10.1001/jamanetworkopen.2023.30754

Data
Data available: No

Additional Information
Explanation for why data not available: This is a study based on published clinical trials
